# Diazoxide-Responsive Hypoglycemia in a Child Receiving Dasatinib for Treatment of B-Cell Acute Lymphoblastic Leukemia

**DOI:** 10.1210/jcemcr/luae219

**Published:** 2024-12-04

**Authors:** Madalin Berra, Juliana Biro, Himala Kashmiri, Mark Daniels

**Affiliations:** Division of Pediatric Endocrinology, University of California Irvine: Children's Hospital of Orange County, Orange, CA 92868, USA; Division of Pediatric Endocrinology, University of California Irvine: Children's Hospital of Orange County, Orange, CA 92868, USA; Division of Pediatric Endocrinology, University of California Irvine: Children's Hospital of Orange County, Orange, CA 92868, USA; Division of Pediatric Endocrinology, University of California Irvine: Children's Hospital of Orange County, Orange, CA 92868, USA

**Keywords:** hypoglycemia, hyperinsulinism, leukemia, tyrosine kinase inhibitor, dasatinib

## Abstract

Tyrosine kinase inhibitors (TKIs) are being used more regularly in treatment regimens for pediatric cancers. They have distinct side effect profiles, including endocrinopathies. Here we present a 2-year-old boy with Philadelphia chromosome–like (Ph-like) B-cell acute lymphoblastic leukemia (ALL) who developed refractory hypoglycemia after using dasatinib. His evaluation was suggestive of hyperinsulinism, and his symptoms were ultimately controlled with diazoxide. There have not been any published data exploring the relationship between TKIs and glycemic profiles in pediatric patients. In adults, there is research indicating that patients using TKIs could experience both hyperglycemia and hypoglycemia. The pathophysiology of these side effects is not well described, nor are the risk factors for development. More research is needed to understand these relationships in general, but particularly in the pediatric population.

## Introduction

Tyrosine kinase inhibitors (TKIs) are a relatively new treatment modality in pediatric cancer. They act on one or multiple receptors and/or protein tyrosine kinases allowing for targeted therapy. The first-generation TKI imatinib was approved in 2003 for use in pediatric chronic myeloid leukemia (CML) and in 2013 for pediatric Philadelphia chromosome and Philadelphia chromosome–like (Ph/Ph-like) B-cell acute lymphoblastic leukemia (ALL). More recently, in 2017, the second-generation TKI dasatinib was approved for use in pediatric CML, and in 2019 for pediatric Ph/Ph-like ALL. There have been additional studies regarding the use of these and other TKIs in pediatric solid tumors such as osteosarcoma, central nervous system tumors, neuroblastoma, thyroid cancer, and gastrointestinal stromal tumors. These medications have several adverse effects impacting the endocrine system, including thyroid dysfunction, metabolic bone disease, adrenal insufficiency, dysglycemia, and others [1].

Here, we present a case of a 2-year-old male patient with Ph-like B-cell ALL who developed diazoxide-responsive hypoglycemia while on dasatinib. To our knowledge, this is the first reported case in the pediatric population.

## Case Presentation

A boy aged 2 years and 4 months, who was on delayed intensification treatment phase for very high-risk Ph-like B-cell ALL, presented to the emergency room for fever and was incidentally found to be hypoglycemic with a blood glucose level on his chemistry panel of 57 mg/dL (3.2 mmol/L). The remainder of his studies were notable for negative urine ketones, normal venous pH of 7.396 (normal range, 7.300-7.400), and chronic stable transaminitis with aspartate aminotransferase (AST) 124 U/L (normal range, 15-46 U/L) and alanine aminotransferase (ALT) 77 U/L (normal range, 4-35 U/L). At home they had noticed increased lethargy with poor oral intake, there was no history of emesis or diarrhea. Given his hypoglycemia, endocrinology was consulted. With further investigation we identified that since his diagnosis of ALL at 18 months, he had experienced intermittent incidental mild hypoglycemia on chemistry panel throughout his treatment course, typically with values 55 to 65 mg/dL (3.1-3.6 mmol/L). He had never received directed therapy or evaluation for his hypoglycemia until the admission. More recently, in the month preceding the admission, he had increased frequency and severity of hypoglycemia with blood sugars ranging from 42 to 68 mg/dL (2.7-3.8 mmol/L) on serum sample. He had no other medical history outside of his oncologic process. His developmental history was notable for a speech delay, but he had otherwise met all developmental milestones on time. There was no exposure to any oral hypoglycemic agents nor insulin in the home.

**Figure 1.**
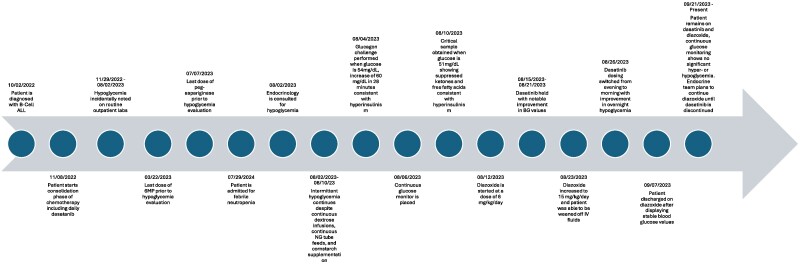
Timeline of clinical events starting at the time of oncologic diagnosis through current status.

During his leukemia treatment he had received chemotherapeutic agents including cytarabine, vincristine, PEG-asparaginase, both intravenous (IV) and intrathecal methotrexate, and 6-mercaptopurine (6-MP). He received glucocorticoids intermittently. He was also on daily dasatinib at a dose of 30 to 40 mg starting approximately 6 weeks into his treatment. At the time of admission, he was on dasatinib 30 mg oral daily and Bactrim prophylaxis 2 days per week; he had last received PEG-asparaginase 3 weeks prior and his last dose of 6-MP was 4 months prior. The timeline summary is presented in Fig. 1.

## Diagnostic Assessment

Over the subsequent days, he continued to have hypoglycemia on point-of-care blood glucose readings, despite the use of dextrose containing IV fluids with a glucose infusion rate up to 9 mg/kg/min. Any attempts to discontinue his dextrose IV fluids resulted in hypoglycemia as low as 38 mg/dL (2.1 mmol/L). A critical sample was obtained when blood glucose was 51 mg/dL (2.8 mmol/L). He had inappropriately low ketones of 0.12 mMol/L (normal range during hypoglycemia ≥ 0.6 mMol/L) and free fatty acids of 0.15 mMol/L (normal range during hypoglycemia ≥ 0.5 mMol/L). Insulin level at this time was 0.32 µIU/mL (1.92 pmol/L, normal range during hypoglycemia < 2 µIU/mL or < 12 pmol/L). After another instance of hypoglycemia, he underwent a glucagon challenge and received 1 mg of glucagon; he had an increase in his blood glucose of 60 mg/dL (3.3 mmol/L) in a period of 28 minutes, which was deemed a positive response. His evaluation for hypothyroidism and adrenal insufficiency yielded normal thyroid function tests and cortisol level. A sample for ammonia level was also drawn which was normal. His examination was significant for thin appearance and negative for any hepatosplenomegaly.

The remainder of his workup was significant for negative diabetes auto-antibodies (islet cell antigen 512, glutamic acid decarboxylase-65, insulin, zinc transporter 8), and a negative Invitae genetic hypoglycemia panel to test for pathogenic gene variants associated with congenital hyperinsulinism.

## Treatment

Given his hypoketosis and low free fatty acids with a positive glucagon response, his diagnosis was most consistent with hyperinsulinism. To gather additional real-time information, we were able to place a continuous glucose monitor to identify his glycemic trends while he was admitted.

His hospital course was complicated by a poor appetite, so initially we attempted to treat his hypoglycemia with cornstarch mixed into formula and administered continuously via nasogastric tube overnight. Despite this, he continued to drop into the mid 50 mg/dL (2.5 mmol/L) range overnight. He also had significant intolerance of tube placement, and he would repeatedly pull the tube out. Due to his suboptimal response to nonpharmacologic interventions, we started diazoxide at a dose of 10 mg/kg/day. Given our review of the literature and possible link of the TKI to hypoglycemia, we also held dasatinib to better understand its relationship to his hypoglycemia. His blood glucose trend improved and after 6 days the dasatinib was resumed. Daytime blood glucose remained in range, but he had redemonstration of overnight hypoglycemia. In response, the dasatinib administration timing was changed from late evening to early morning, resulting in resolution of overnight hypoglycemia.

## Outcome and Follow-Up

The patient was discharged on diazoxide and has remained stable on this regimen without significant hypoglycemia or hyperglycemia over the last 5 months. He continues to use a Dexcom G6 continuous glucose monitor. He has not needed any diazoxide dose titrations. Our plan is to continue diazoxide therapy until the end of his cancer treatment unless he develops significant hyperglycemia prompting a decrease in his dose.

## Discussion

Precision medicine allows for more directed care, often with improved disease outcomes, although not without a side effect profile. TKIs have a particular association with the development of endocrinopathies. The most common is thyroid dysfunction, but other endocrinopathies include adrenal insufficiency, growth hormone deficiency, hypogonadism, and abnormal bone mineral density [1]. Of interest to our case is the impact on glycemic profile.

There have been multiple case reports describing adult patients with type 2 diabetes mellitus who experienced decreasing insulin requirements, improving glycated hemoglobin (HbA1c) levels, and/or hypoglycemia after receiving dasatinib [2‐4]. Multiple retrospective chart reviews have suggested the same trend of improved diabetes control and/or decreasing insulin needs in patients with type 2 diabetes receiving TKIs [5, 6]. Although most reports are focused on patients with diabetes, one retrospective chart review gathered data regarding glycemic profile from 80 patients with and without diabetes who had received a TKI (imatinib, dasatinib, sunitinib, or sorafenib) [7]. The average glucose trend declined for patients receiving any of these TKIs, and almost half of the patients with diabetes were able to completely discontinue their insulin therapy [7]. The dasatinib group was the smallest, consisting of only 8 patients, but it had the largest average glucose decline of 52 mg/dL (2.9 mmol/L) [7]. Less commonly, there have been reports of increased rate of hyperglycemia and metabolic syndrome associated with TKI use [8]. There have been no previously published reports of dasatinib-associated hypoglycemia in pediatric patients, and no retrospective reviews on the subject.

Patients undergoing chemotherapy often receive multiple medications with different side effect profiles, and it can be difficult to determine a causal relationship between a medication and a side effect. In our patient's case we feel confident, after weeks of observation, that dasatinib was the driving factor in his hypoglycemia. We considered PEG-asparaginase as a causative agent, as this has been described in case reports [9]. Most reports of PEG-asparaginase associated hypoglycemia have normalization of their blood glucose values by 4 to 5 weeks after receiving the medication [9], whereas our patient had persistence of the symptoms 8 weeks after his last dose. We also considered 6-MP as a possible causative agent, as hypoglycemia is a rare but well documented side effect of this medication in young children. However, given that the patient was 4 months removed from his last dose of 6-MP we believed this to be unlikely, as hypoglycemia due to 6-MP is typically resolved within 4 months of completing this therapy [10]. In addition, reports of 6-MP-induced hypoglycemia note that patients have significant ketosis, whereas our patient had low ketone levels during hypoglycemia [10‐12]. Further evidence of dasatinib as the causative agent was the fact that we saw improvement in hypoglycemia during a 6-day pause in dasatinib usage, with subsequent worsening hypoglycemia after resuming the medication. Additionally, he had intermittent, though less severe, hypoglycemia noted at many points in his historical treatment course with no temporal relationship to PEG-asparaginase or 6-MP.

Although he did not have an elevated insulin level, the preponderance of evidence relating to his hypoglycemia was most consistent with hyperinsulinism as the mechanism. This was supported by inappropriately low ketones and free fatty acids at the time of hypoglycemia as well as a positive response to glucagon stimulation with a blood glucose rise of 60 mg/dL (3.3 mmol/L) within 30 minutes. Additionally, his success with diazoxide therapy is consistent with an insulin-mediated process.

The pharmacological explanation for this side effect is not yet defined. Dasatinib's main therapeutic action in Ph/Ph-like ALL is inhibition of BCR-ABL, but it also acts on multiple other tyrosine kinases and receptor tyrosine kinases, including Src family kinases, c-Kit, platelet-derived growth factor receptor, and others [13]. The kinase c-Kit plays a role in embryonic development of islet cells and ongoing function in mature beta cells. Mouse studies have indicated that increased c-Kit expression leads to increased insulin secretion, so it would follow that c-Kit inhibition would more likely cause hyperglycemia than hypoglycemia [14]. There may be a relationship between c-Kit inhibition and overall beta-cell dysfunction, but this does not seem like a plausible explanation for hyperinsulinism.

This case is the first to suggest that hypoglycemia secondary to hyperinsulinism is a potential side effect in pediatric patients using dasatinib, and that it may be successfully treated with diazoxide. Further research is needed to characterize the metabolic side effects of tyrosine kinase inhibitors in pediatric patients, and ultimately to better understand the mechanisms leading to these effects.

## Learning Points

- The use of TKIs is increasing in the treatment of pediatric cancers, these medications are associated with endocrinopathies.
- Available literature suggests that dasatinib and other TKIs can cause dysglycemia, most commonly hypoglycemia. There have been several cases describing this in both previously healthy adult patients and those with a history of type 2 diabetes. There have been no published cases or reviews describing this in pediatric patients.
- The mechanism of hypoglycemia secondary to dasatinib is not well described, but as seen in this case, it may be due to an insulin-mediated process.

## Contributors

M.B., J.B., H.K., and M.D. were involved in the diagnosis and management of this patient; M.B. and J.B prepared the initial manuscript draft; H.K. and M.D. completed editing. All authors contributed to the research. M.B. prepared the submission. J.B. created figures. All authors contributed to submission revisions.

## Data Availability

Data sharing is not applicable to this article as no datasets were generated or analyzed during the current study.

## Abbreviations

6-MP: 6-mercaptopurine
ALL: acute lymphoblastic leukemia
IV: intravenous
Ph: Philadelphia chromosome
TKI: tyrosine kinase inhibitor
